# Notes from Private Practice

**Published:** 1878-01

**Authors:** E. R. Willard

**Affiliations:** Wilmington


### Clinical Reports.

### NOTES FROM PRIVATE PRACTICE.

*A Case of Ovariotomy.*

Mrs. S. B. D. L.—Mother of two children, of good constitution and fair health, began to notice in the summer of 1874, an enlargement of the abdomen. During the fall she was seized with a severe attack of violent paroxysmal pain, which confined her to her bed for several days, after which the tumor in the abdomen could not be felt. In the summer of 1875 it began to enlarge, and she called upon Dr. E. R. Willard. He then diagnosticated an ovarian tumor, about four inches in diameter. After this the abdomen slowly increased in size, until the 28th of August, 1875, when she was seen by Dr. A. W. Heise, of Joliet, Ill., who advised paracentesis, which was performed by Dr. Merriman, and twelve pounds of albuminous fluid evacuated. Four weeks later, the sac having rapidly refilled, and the pressure becoming great, it was thought best to again relieve the patient by tapping, which was done on the 30th of September, and twenty pounds of very thick albuminous fluid removed.

On Oct. 20th, the sac having nearly refilled, her father and husband said that if Dr. E. R. Willard, in connection with Dr. Merriman, of this place, would undertake the removal of the tumor, they "would be entirely satisfied, let the result be what it might." The patient had been taking the elixir of iron and bark for several weeks; this was ordered to be continued.

The bowels were moved on the 25th, by a dose of senna and magnesium sulphate.

At 12 m., Oct. 26th, she was given brandy. At 1:30 p. m., etherization was commenced by Dr. G. E. Willard, in a room brought to a temperature of 32° C. The patient having been etherized, an incision reaching from near the umbilicus to the symphysis was made, the peritoneal cavity entered, and the peritoneum divided between the fingers. The tumor was then seized with a pair of duck-bill forceps, the patient partially turned upon her left side, an opening made in the tumor with a bistoury, and the fluid evacuated as quickly as possible by enlarging the opening and introducing the hand into the tumor and breaking up the cysts from the inside, thereby preventing the fluid from coming in contact with the peritoneum.

The adhesions were slight, and easily broken up. After drawing the tumor through the abdominal parietes, the pedicle was secured with Spencer Wells' clamp, and severed; the abdomen sponged with fresh, warm, carbolized water (the hands, sponges, and instruments were all dipped in carbolized water before use about the patient), and the wound closed with silk sutures according to the plan of Spencer Wells—pledgets of cotton, dipped in carbolized oil, were placed upon the wound and around the clamp; when the entire abdomen was covered with cotton wool two inches in thickness. A flannel bandage was then pinned moderately tight over the whole, and the patient put to bed.

The weight of the entire tumor, fluid and solid, removed in the operation, was thirty pounds. Time occupied in the operation, twenty-five minutes.

Thirty minutes after the operation, pulse, 86; temperature, 36°†; 6 p. m., pulse, 88; temperature, 37½°; directed beef essence every four hours.

Oct. 27th, 3:30 a. m., pulse, 100; temperature, 37 8-10°; urine drawn every four hours; 8 a. m., pulse, 100; temperature, 37 8-10°; 12:30 p. m., pulse, 96; temperature, 38°; 6 p. m., pulse, 100; temperature, 38¼°.

Oct. 28th, 4 a. m., pulse, 100; temperature, 37½°; 9 a. m., pulse, 100; temperature, 37 6-10°; some nausea; 6 p. m., pulse,

† The temperature in this case is stated throughout as registered on the Centigrade scale.

114; temperature, 38 1-10°; nausea increasing; has retained only one-half pint of milk in the last twelve hours; vomited several times in the course of the day; ordered five grains of citrate of iron every six hours, and one-sixteenth of a grain of morphine every six hours alternately with the iron; gave two ten-grain doses of the sub-carbonate of bismuth, at an interval of thirty minutes, to correct the acidity of the stomach.

Oct. 29th, 8 a. m., pulse, 105; temperature, 37 6-10; has hiccough every ten to twenty minutes; 1 p. m., pulse, 105; temperature, 37 8-10°; has had one-eighth grain morphine at 1 a. m. and 8 a. m; 6 p. m., pulse, 108; temperature, 37 3-10; gave two grains of quinine.

Oct. 30th, 8 a. m., pulse, 94; temperature, 37°; gave hypodermic injection of one-eighth grain of morphine at 11 p. m. last night; hiccough less; retains solid food; passed urine naturally; gave one pint of warm water as an injection, with a few drops of carbolic acid 95 per cent.; has taken two grains of quinine every four hours the past twenty-four hours; 6 p. m., pulse, 92; temperature, 37 6-10°; gave one-eighth grain of morphine hypodermically.

Oct 31st, 8 a. m., pulse 92, temperature 37 6-10°; hypodermic injection of $\frac{1}{8}$ gr. morphine; sickness increasing; 6 p. m., pulse 105, temperature 37 7-10; ordered lime water and milk as an exclusive diet.

Nov. 1st, 8 a. m., pulse 95, temperature 37 6-10°; nausea and hiccough relieved; added mutton broth and crackers to lime water and milk; no medicine; 6 p. m., pulse 104, temperature 37 7-10°.

Nov. 2d, 8 a. m., pulse 103, temperature 37 2-10; some appetite, fish and potatoes added to diet; 6 p. m., pulse 107, temperature 37 7-10°.

Nov. 3d, 8 a.m., pulse 97, temperature 37°; removed all the sutures, clamp not sloughed off; 6 p. m., pulse 105, temperature 39°; appetite good.

Nov. 4th, 8 a. m., pulse 120, temperature $37\frac{1}{2}$°; 6 p. m., pulse 125, temperature 39°.

Nov. 5th, 8 a. m., pulse 120, temperature $37\frac{1}{2}$°; clamp removed; wound closed entirely by first intention; 6 p. m., pulse 120, temperature 37°; bowels moved by injections.

Nov. 6th, 8 a. m., pulse 110, temperature 37 3-10; 6 p. m., pulse 104, temperature 37 8-10.

Nov. 7th, 8 a. m., pulse 100, temperature 37°; patient feels well, wants to get out of bed, thinks she is able to be up; ordered 5 gr. doses of bromide of potassium, for a slight smarting sensation after passing water.

Nov. 8th, 8 a. m., pulse 110, temperature 37½; 1 p. m., restless, gave ½ gr. of svapnia; 6 p. m., pulse 118, temperature 38°; gave a cathartic of castor oil.

Nov. 9th, 8 a. m., pulse 125, temperature 37½°; 6 p. m., pulse 124, temperature 38½°; bowels moved at 3 p. m.

Nov. 10th, 8 a. m., pulse 112, temperature 37½; ordered 4 gr. doses of cinchonidia every four hours.

Nov. 11th, 8 a. m., pulse 102, temperature 37½.

Nov. 12th, 8 a. m., pulse 110, temperature 37°; is permitted to sit up two or three hours in the day. After this the patient rapidly improved, and is now quite out of danger.

E. R. Willard, M. D.

Wilmington, November, 1877.

---

